# The Difficult Integration between Human and Animal Studies on Emotional Lateralization: A Perspective Article

**DOI:** 10.3390/brainsci11080975

**Published:** 2021-07-23

**Authors:** Guido Gainotti

**Affiliations:** Institute of Neurology, Catholic University, Largo Agostino Gemelli 8, 00168 Rome, Italy; guido.gainotti@unicatt.it; Tel.: +39-06-35501945; Fax: +39-06-35501909

**Keywords:** laterality of emotions in animals, models of emotional lateralization, right lateralization of sympathetic components of emotions, asymmetries in experience and expression of emotions

## Abstract

Even if for many years hemispheric asymmetries have been considered as a uniquely human feature, an increasing number of studies have described hemispheric asymmetries for various behavioral functions in several nonhuman species. An aspect of animal lateralization that has attracted particular attention has concerned the hemispheric asymmetries for emotions, but human and animal studies on this subject have been developed as independent lines of investigation, without attempts for their integration. In this perspective article, after an illustration of factors that have hampered the integration between human and animal studies on emotional lateralization, I will pass to analyze components and stages of the processing of emotions to distinguish those which point to a continuum between humans and many animal species, from those which suggest a similarity only between humans and great apes. The right lateralization of sympathetic functions (involved in brain and bodily activities necessary in emergency situations) seems consistent across many animal species, whereas asymmetries in emotional communication and in structures involved in emotional experience, similar to those observed in humans, have been documented only in primates.

## 1. Introduction

In a special issue of *Brain Sciences* dealing with “Emotions and the Right Hemisphere”, it could perhaps be appropriate to express a few considerations on a theme which is usually ignored in this area of research, in spite of its general interest, namely, the problem of the poor interaction between human and animal studies on emotional asymmetries. From a historical point of view, this lack of communication between authors interested in human and animal sciences probably stems from the fact that, given that language is considered as a hallmark of our species [1], hemispheric asymmetries have been considered as a uniquely human feature since the discovery of the left lateralization of language. Several authors, see [2,3,4], have even suggested that handedness and cerebral asymmetry resulted from some genetic mutation at some point after the split of the hominins from the other great apes. Over the past decades, however, this idea has been challenged by an increasing number of studies describing hemispheric asymmetries for various behavioral functions in several nonhuman species. The main view of these studies was summarized in a book by Bradshaw and Rogers [5], which provided a thorough review of behavioral and morphological asymmetries in a wide variety of species, with a concentration on birds, rodents, and primates. In contrast with Corballis’ [2] views, assuming that human language is a uniquely human property that has evolved quite recently, Bradshaw and Rogers [5] argued that there have long been different forms of functional asymmetry, and that the present state of affairs in homo sapiens is the final product of this coevolution. Irrespectively of the reasons for this debate, an aspect of animal lateralization that has attracted particular attention has concerned the hemispheric asymmetries for emotions, probably because most investigations dealing with this subject were based on observational, behavioral or physiological measurements that are particularly appropriate for the study of animal behavior, see [6,7]. These investigations have revealed important homologies between human and animal asymmetries for various aspects of emotional behavior, but many problems concerning theoretical and factual issues remain open in this area of research. Some of these problems concern the general differences between the studies of hemispheric asymmetries in animals and humans, whereas other problems concern the difficulty of integrating human and animal studies in the more specific area of hemispheric asymmetries for emotions.

In this perspective article, I will start from a short illustration of the general factors that have hampered the integration between human and animal studies on hemispheric asymmetries, to pass to an elucidation of factors that have more specifically failed to establish a dialogue between human and animal studies on the laterality of emotions. I will then pass to a comparative analysis of components and stages of the processing of emotions, to distinguish those which point to a continuum between humans and many animal species, from those which seem more problematic from this point of view.

## 2. General Factors That Have Hindered the Integration between Human and Animal Studies on Different Aspects of Hemispheric Functional Asymmetries

A first problem that has hampered the integration between human and animal studies has resided in the very large number of animal species, belonging to different levels of the phylogenetic evolution, that have been taken into account in these investigations. Some studies have been, indeed, limited to a comparison between humans and nonhuman primates (see [8,9,10,11] for review), but in most other papers, the study of hemispheric asymmetries has extended to all nonhuman vertebrates (see [12,13,14,15] for review). Still other investigations have focused attention on lower vertebrates (see [16] for review) or even on invertebrates (see [17,18] for review), suggesting that invertebrates with complex brains, such as arthropods and cephalopods, share the attribute of lateralization with vertebrates. Now, even if MacNeilage et al. [14] have assumed that all hemispheric specializations might have evolved from a basic lateralization pattern, common to all vertebrates and even to invertebrates, it seems unlikely that the same asymmetry of function may be equally adaptive in different brains and in different environmental and social contexts, such as those typical of insects, fishes, birds and mammals. It is therefore unclear whether the observed hemispheric asymmetries should be interpreted as reflecting a basic homology, or parallel but independent evolutionary histories. More generally, it must be noticed that human and animal studies on hemispheric asymmetries for emotions have been developed as rather independent lines of investigation, without attempts to integrate these research domains. 

A second important reason that has hampered this integration has been the different audiences addressed by human and animal studies. Human investigations are, in fact, usually published in neuropsychology journals, such as *Neuropsychologia*, *Cortex*, *Neuropsychology*, *Trends in Cognitive Sciences, Neuropsychology Reviews* and so on, whereas animal investigations are usually published in animal behavior journals, such as *Animal Behaviour*, *Applied Animal Behaviour Science*, the *Journal of Animal Sciences* and so on. Actually, both human and animal studies are also published in journals that address important issues on neuroscience, such as *Neuroscience and Biobehavioral Reviews*, *Brain Research*, *Behavioural and Brain Research*, and *Laterality*. However, since the interest of researchers is seldom attracted by papers that do not belong to their specific area of research, this has not been sufficient to establish a dialogue between human and animal disciplines. An important factor that has contributed to discouraging this dialogue lies in the fact that, due to the diverging brain organizations of different animal species, methods of study of behavioral asymmetries have been inconsistent between human and animal studies (see [19,20,21] for recent reviews of methods used in the study of animal lateralization). Now, since a good knowledge of the advantages and limitations of different methods is necessary to make a critical assessment of a research paper, scholars of human asymmetries have rarely been attracted by results obtained in the study of animal lateralization.

## 3. Factors That Have Specifically Failed to Establish a Dialogue between Human and Animal Studies on Hemispheric Asymmetries for Emotions

### 3.1. Behavioral Patterns Considered as ‘Emotional’ in Human and in Animal Studies 

An important reason that has impeded the integration between human and animal studies on the specific problem of hemispheric asymmetries for emotions has been the limited overlap between the behavioral patterns considered as ‘emotional’ in human and in animal studies. In fact, even if the exact nature and categorization of emotions remains controversial, most scholars of human emotions, see [22,23,24,25,26,27], agree that emotions are short and coordinated sets of physiological, behavioral, and expressive responses to the situational antecedents typical of each emotion. This definition is particularly appropriate to the construct of “basic emotions” (happiness, sadness, fear, anger, surprise and disgust), which correspond to a small number of innate and hard wired behavioral patterns, triggered by specific survival related events. Basic emotions are usually distinguished from “social emotions” (e.g., embarrassment, guilt, shame, empathy, and pride), that are subtended by social norms and are tightly linked with the development of social cognition. In any case, human emotions are distinguished from basic survival related appetitive behaviors (such as those concerning food and sex), whereas this distinction is not made by scholars of animal emotions. Sex, indeed, constitutes an important part of investigations dealing with emotional asymmetries in various invertebrate and vertebrate species, such as gastropods [28], insects [29], fishes [30], amphibians [31], birds [32,33,34], and mammals (where they have concerned dogs [35], rhesus macaques [36], Mongolian gerbils [37] and mouse lemurs [38]). Even more significant is the number of papers dealing with side preferences for food rewards (attacking prey), because this subject has been investigated in spiders [39], ants [40], fishes [41,42], amphibians [43], reptiles [44], birds [32,45,46,47] and mammals. Within this vertebrate group, a side preference for food rewards has been studied in dogs [35] and in primates [48,49,50,51]. In all these studies, the evaluation of results obtained was complicated by the different procedures used to assess asymmetries in the feeding and sexual appetitive behaviors of animals which have different bodies and different neural organizations. Furthermore, Marchant and McGrew [51], in their thorough discussion of the meaning of the term ‘handedness’ in chimpanzees, stressed the methodological difficulties of the comparative use of this term, even when comparison is made between phylogenetically near animal species, such as chimpanzees and humans.

### 3.2. Models of Hemispheric Asymmetries for Emotions Transposed from Human to Animal Studies

A second important source of difficulty in the integration between human and animal studies has been that three different models of hemispheric asymmetries (usually called the “right hemisphere hypothesis”, the “emotional valence hypothesis” and the “approach–withdrawal hypothesis”) have been advanced to explain results of clinical and experimental studies for emotions in man, and that these models have been transposed from human to animal studies. 

*The “right hemisphere hypothesis”* was originally proposed by Gainotti [52], analyzing the patterns of emotional behavior shown by right and left brain damaged patients (BDPs) confronted with difficulties during a neuropsychological examination. This author noted that (aphasic) left BDPs showed a “catastrophic reaction” when confronted with frustrating attempts at verbal expression, whereas right BDPs showed “indifference reactions” during similar frustrating situations. Gainotti considered the “catastrophic reactions” of left BDPs as a dramatic, but psychologically appropriate, form of reaction to a catastrophic event, whereas he viewed as quite abnormal the “indifference reactions” of right BDPs. He therefore assumed that the abnormal emotional reaction of right brain damaged patients could reflect the major involvement of the right hemisphere in emotional functions, just as the language disturbances of left brain damaged patients reflect the left hemisphere dominance for language. *The “emotional valence hypothesis”* was analogously proposed by authors [53,54,55], who had observed, respectively, a “catastrophic reaction” and a “euphoric reaction” in patients submitted to left and right intracarotid Amytal injection. These authors interpreted “catastrophic reactions” and “euphoric reactions” as due to the inactivation neural mechanisms underpinning positive and negative emotions and located, respectively, in the left and right hemispheres.

In following years, these different models were checked by experimental investigations that studied the comprehension and expression of emotions in normal subjects and in right and left brain damaged patients. Taken together, the results of both experiments conducted in normal subjects and those carried out in patients with unilateral brain lesions were much more consistent with the predictions of “right hemisphere dominance” than with those of the “valence hypothesis” (see [56,57,58,59] for review). However, some investigations conducted in normal subjects by Schwartz et al. [60], Reuter-Lorenz and Davidson [61], Reuter-Lorenz et al. [62] and Natale et al. [63] suggested an advantage of the right hemisphere in the recognition of negative emotions and of the left hemisphere in the recognition of positive emotions. 

At variance with the “right hemisphere hypothesis” and the “emotional valence hypothesis”, which had been raised by clinical data and checked by experimental investigations conducted in normal subjects and in brain damaged patients, *the “approach–withdrawal hypothesis”*, was proposed by Davidson [64,65,66] based on electroencephalographic (EEG) data recorded at the level of the frontal lobes in normal subjects, during the expression of positive and negative emotions [67,68]. Since, according to Davidson [64,65,66], these asymmetries are not related to the valence but to the motivational system engaged by the emotional stimulus, he proposed that the left prefrontal cortex (PFC) might be involved in a system-inducing approach to appetitive stimuli and the right PFC in a system promoting withdrawal from aversive stimuli. An important drawback of this model consisted in its difficulty to account for the clinical and experimental data that had suggested the previous models of hemispheric asymmetries for emotions. Furthermore, an even more radical objection was expressed by authors who criticized both the relation between PFC and approach/avoidance tendencies, see [69], and the use of frontal EEG asymmetries to assess emotion or motivation, see [70,71].

All these models of hemispheric asymmetries for emotions have been used in animal studies. Thus, Quaranta et al. [72] and Siniscalchi et al. [73] maintained that asymmetric tail-wagging responses by dogs to different emotive stimuli were consistent with the approach–withdrawal hypothesis. This claim was due to the fact that stimuli that elicited approach tendencies, such as seeing the dog’s owner, were associated with a higher amplitude of tail wagging movements to the right side (left brain activation), whereas stimuli that elicited withdrawal tendencies, such as seeing a dominant unfamiliar dog, were associated with higher amplitude of tail wagging movements to the left side (right brain activation). 

This model was, however, questioned by other authors (see [30,32,35,74,75,76]) who reported in different animal species a general pattern of right hemisphere dominance in processing fear and aggression (of predominantly negative emotional valence), and of left hemisphere dominance in processing responses to sex or food rewards (of positive emotional valence). These authors claimed that the “emotional-valence hypothesis” (rather than the “approach–withdrawal hypothesis”) is supported by these results, because both fear and anger (underpinned by the right hemisphere) are negative emotions, but fear is associated with withdrawal behavior, whereas aggression is associated with approach behavior. Thus, in a careful review of emotion lateralization in animals, Lelived et al. [77] focused on five major emotional contexts that have been studied with **respect** to lateralization, namely those associated with fear/anxiety, aggression, sex, responses to food rewards and positive social interactions. The context “fear/anxiety” included studies that focused on several situations, such as the presence of a predator (simulation) or facing a potentially life threatening situation. The context “aggression” included studies that observed agonistic conspecific interactions, excluding studies that dealt with interspecific predatory attacks. The context “sex” included studies that observed sexual behavior or presented a sexual stimulus. The fourth context, “responses to food rewards”, included studies in which animals responded to the presence of food (predation, food discrimination and food observation) and the final context “general positive social situations”, included studies on play, contact, and observation of conspecifics. The authors concluded that, although a clear overview was not always found for each class, some general patterns could be discerned. For instance, fear/anxiety and aggression seemed to be predominantly processed by the right hemisphere in most classes, excepting fishes. Responses to food rewards seemed to be predominantly processed by the left hemisphere, except in primates, whereas no clear pattern could be found for sex and positive social situations. 

It must be noted, however, that in primates the contrast between a left hemispheric dominance for positive emotions (food rewards, sex and positive social situations) and a right hemispheric dominance for negative emotions (fear and aggression) has not been found. Furthermore, most studies dealing with asymmetries in emotional expression in this phylogenetically advanced class of mammals, see [9,36,49,78,79,80,81,82,83], have suggested a right hemisphere dominance across negative and positive emotions, which appears consistent with the right hemisphere model of emotional asymmetry. 

## 4. Comparative Analysis of Components and Stages of Emotional Processing That Could Contribute to a Better Integration between Human and Animal Investigations

In human studies, emotions are usually considered as a multicomponent adaptive system; namely, as an integrated set of components that allow the organism to face a partially unpredictable environment and select the most appropriate plans of action from among those available. From this point of view, similarities and differences exist between the emotional and the cognitive systems, because both are adaptive systems that must orient attention toward the relevant stimuli, compute their meaning, and select the most appropriate response patterns, but their goals are different. The emotional system is, indeed, regarded as an emergency system, able to interrupt ongoing activity to rapidly select a new operative scheme, whereas the cognitive system is viewed as a more complex, conscious and controlled system which, having the drawback of requiring more time to carry out its work, is not very appropriate for emergency situations Authors interested in human emotions have tried to identify components and staging of this emergency emotional processing, as well as the brain structures involved in each component of this process. As a result of this effort, these authors have also tried to identify the hemispheric asymmetries that could be detected in each of these stages. A similar focalization on the components of emotional processing has less frequently been attempted by authors interested in animal emotions, who have rather centered their attention on the most appropriate methods of study of the behavioral asymmetries, see [19,20,21], and on the basic mechanisms underlying these asymmetries at the individual or the species level, see [84]. From a rather simplistic point of view, it could be said that the main components of the emotional process consist of: (a) a quick computation of poorly processed sensory data, sufficient to decide if an external situation has a pleasant or dangerous meaning; (b) an integration of these sensory data within the central nervous system to select the most appropriate behavioral response; (c) an automatic instantiation of action schemata that must include expressive–communicative components, postural changes, bodily movements and a sizeable recruitment of the autonomic nervous system. The expressive components of the response should serve to communicate the existence of a survival related or behaviorally relevant situation to the other members of the group; postural changes and bodily movements tend to initiate the selected action schema, and the recruitment of the autonomic nervous system helps to mobilize all the energies necessary to solve the emergency situation. This schematization is, however, simplistic, because it describes emotions as purely cerebral events, whereas the (human or animal) body plays an equally important role in each stage of the emotional process. Since James’ [85] proposal, assuming that emotion is a perception of bodily changes, it has, indeed, been suggested that recognition of the personal relevance of a given situation is a necessary, but not sufficient, condition for the origination of emotions. Drawing on James’ [85] proposal, it has been proposed that emotion is generated when the amygdala, which makes a quick computation of poorly processed sensory data, sends a message to the hypothalamus [86,87] and peri-aqueductal gray/PAG [88,89], which, in turn, forward this message to the internal organs of the body and to skeletal and expressive muscles through different sections of the vegetative and somatic nervous system. The response of these bodily parts is sent back to the brain, and in particular to the insular cortex, which receives interoceptive inputs from the whole body in its posterior parts, and has bidirectional connections in its anterior parts with other cortical lobes, integrating lower level interoceptive and homeostatic information with higher level cognitive representations [90]. Now, there are reasons to propose that these reflections on these bodily components of emotions may be of some relevance for the development of fruitful interactions between researchers interested in human and animal asymmetries in emotional behavior. The first (theoretical) reason is that bodily reactions should be a more basic mechanism than the comprehension or expression of emotions and should, therefore, be present even at more primitive levels of animal evolution. Now, since, in humans, different kinds of behavioral, functional or anatomical asymmetries have been detected for each stage of this interaction between the brain and the body, these asymmetries could also be present in animals. The second (more factual) reason is that while asymmetries in emotional communication **equivalent** to those observed in humans have been documented only in primates (e.g., [9,36,49,79,80,82,83], see reviews in [10,11]), and in few other social mammals, such as domestic pigs [15], asymmetries concerning the brain–body interactions have also been documented in animals belonging to more primitive levels of phylogenetic evolution. Furthermore, it must be noted that in animal behavior research, a distinction must be made between a cue and a communicatory signal, because an outward behavior stemming from an emotional response to a situation does not need to serve a communicative meaning. This implies that this behavior has not necessarily been evolutionarily selected for communication purposes. For all these reasons, it is possible that a more detailed description of asymmetries discovered in bodily components, rather than in communicative aspects of emotions, may facilitate the integration between human and animal investigations on emotional asymmetries.

### 4.1. Asymmetries in the Evalutation of the Emotional Stimuli in the Right and Left Amygdala

Many authors, see [87,91,92] have proposed that the amygdala could be the structure where external stimuli are evaluated in terms of their emotional significance. An important problem, raised on this subject by Morris et al. [93,94], concerns the possibility that the right and left amygdala may play a different role in the evaluation of emotional stimuli. In a first study, Morris et al. [93] used PET to study the mechanism of an unconscious form of emotional learning in which an aversively conditioned masked emotional face elicited an unconscious emotional response. These authors showed that the masked presentation of the emotional face provoked a significant neural response in the right, but not the left, amygdala, whereas the unmasked presentation of the same stimulus enhanced neural activity more in the left than in the right amygdala. In a second study, the same authors [94] tried to evaluate if the distinction between a cortical and a subcortical route of emotional processing (proposed by Papez [95] and documented by LeDoux et al., [96] could help to clarify the mechanisms through which these different forms of unconscious and conscious emotional learning can be mediated. They observed an increased correlation between the right amygdala and structures of the subcortical route (pulvinar and superior colliculus) during the unconscious (masked) presentation of conditioned emotional stimuli. On the contrary, no masking dependent correlation changes were observed between the same subcortical structures and the left amygdala. Morris et al. [94], therefore, concluded that emotionally laden stimuli can be processed without conscious awareness by a right hemisphere subcortical pathway, mediating unconscious emotional learning. Further investigations by Nomura et al. [97], Williams et al. [98], Pegna et al. [99], Hung et al. [100], Liu et al. [101] and Tetereva et al. [102], confirmed that the amygdala processes threat related information through a right fast subcortical route and a left slower cortical feedback mechanism (see [103] for a more detailed description of these investigations).

If we pass from human to animal studies, we can say that McFadyen [104] has recently reviewed the evidence concerning the existence across species of a subcortical route to the amygdala similar to that investigated in humans and that a visual subcortical pathway that transmits threat signals to the amygdala and directly triggers fearful behavior has been identified in mice [105,106], mouse [107] and primates [108,109]. However, no study, to our knowledge, has investigated possible asymmetries between the subcortical routes transmitting threat signals to the right and left amygdala.

### 4.2. Hemispheric Asymmetries for the Vegetative Components of Emotions

It is universally acknowledged that a sizeable recruitment of the autonomic nervous system is a marker of emotions, and that the vegetative activation is particularly evident for emotions provided of a high survival value, such as fear and anger. This recruitment of the autonomic nervous system is triggered by messages sent by the amygdala to the hypothalamus and peri-aqueductal gray, and is mainly responsible for the bodily changes that are observed during the processing of emotional stimuli and the development of the emotional response. In particular, the sympathetic activation that can be induced by these messages acts as a strong determinant of the efficacy of the behavioral response because it prepares the whole organism to action, allowing it to respond quickly and strongly to emergency situations. The sympathetic system synergistically acts on the heart (enhancing its rate and force of contraction and thus increasing the availability of oxygen and glucose at the tissue level) and on blood vessels, dilating arteries which perfuse brain structures and skeletal muscles (selectively involved in emergency responses) and constricting those perfusing gastrointestinal organs. Analogously, the sympathetic activation increases oxygen uptake (dilating the bronchioles at the lungs level), and breaks down glycogen stores in the skeletal muscles, with an immediate release of consumable glucose. Finally, it acts at the eye level with a dilation of the pupil, which increases the amplitude of the peripheral visual field. On the contrary, the parasympathetic nervous system restores the organism after the stressful situation, decreasing respiration and heart rate and increasing digestion and glycogen formation. Several sources of evidence, obtained by studying the lateralization of autonomic activities in normal subjects and in patients with unilateral brain damage, have consistently suggested an asymmetrical representation of the sympathetic and parasympathetic activities in the human brain. Gainotti [110] has recently reviewed this problem, analyzing results obtained along several clinical and experimental lines of research which included: (a) the cardiac effects of unilateral brain lesions or unilateral pharmacological brain inactivation, (b) changes in heart rate and blood pressure during electrical stimulation of the right and left insular cortex, (c) the psychophysiological correlates of the selective emotional stimulation of the right and left hemispheres in normal subjects. All these investigations documented a leading role of the right hemisphere in the modulation of sympathetic activities, whereas the lateralization of parasympathetic functions was more controversial. Insular stimulation data, did, indeed, suggest that sympathetic cardiovascular regulation is mainly a right insular function, whereas parasympathetic cardiac neural regulation predominates in the left insula, but data obtained during selective emotional stimulation of the right and left hemispheres in normal subjects seemed to point to a right hemisphere dominance for parasympathetic, as well as for sympathetic, activities. It seemed, therefore, safe to conclude that a difference exists between the strong right lateralization of sympathetic activities and the weak left lateralization of parasympathetic activities. 

In animal studies, the right hemisphere has often been associated with the response of the sympathetic nervous system to emergency situations, novel stimuli, fear and aggression, see [21,111,112,113,114,115]. Several motor and sensory markers of right hemisphere prevalence have been used in these studies. Thus, motor markers range from the direction of orientation towards the opponent, with approach on the left side indicating right hemisphere control of “fight or flight” behavior, see [42,111,116,117] to the asymmetric tail-wagging responses by dogs to different emotive stimuli, see [35,72,73]. Analogously, sensory markers range from hearing or visual asymmetries, to the lateralized processing of olfactory emotional stimuli. In the first case, a right hemisphere preeminence is suggested by head orienting to the left side (contralateral to the activated right hemisphere) in response to alarming or emotionally arousing stimuli, see [115,117,118,119]. In the case of olfactory emotional stimuli, a right nostril preferential use during sniffing arousing stimuli was found in both dogs and in, see [21,35,120,121], because in mammal brains odor information is processed ipsilaterally. Not all these behavioral asymmetries have been, however, clearly and consistently explained. Thus, controversies about motor asymmetries have concerned the individual or population level of the lateralization as well as the direction/strength of the motor bias and the effect of this lateralization on fight outcome in different animal species, such as baboons [122], deer [123], horses [124], and pigs [125]. In spite of these inconsistencies, these investigations suggest a continuum between human and animal studies with respect to the issue of the right hemisphere prevalence in the modulation of sympathetic activities 

### 4.3. The Contribution of the Right and Left Insular Cortex to the Emotional Experience

We have seen, in a previous part of this section, that the insular cortex is informed, through the spinothalamic tracts and the afferent branches of the vegetative system, of the effects provoked at the level of visceral and somatic body parts by the emotional stimuli automatically processed by the amygdala and forwarded to PAG and the hypothalamus. Drawing on the connectivity pattern of the insular cortex that allows for the integration between lower level homeostatic representations and higher level cognitive functions [125], Craig [90,126,127] proposed a posterior–anterior gradient in the insular cortex. According to this proposal, physical features of interoception should be processed in the posterior insula, whereas the integration of interoception with cognitive and motivational information should be refined in the anterior insular cortex (AIC). The emotional experience information, which is generated by this integration between lower level (bodily) and higher level (cognitive), is probably shared by animals [7], as was anticipated by Darwin [128], who took it for granted that animals not only behave emotionally but experience those emotions as well. Obviously, there are different levels of emotional experience, whose more conscious aspects can be considered as an important component of the process that leads to the formation of emotional representations [129]. The latter result, in fact, from the convergence of data concerning the emotionally laden situation, the concomitant experience, the associated response and the personal implications of this response. An important overlap exists, therefore, between the construct of emotional experience and Damasio’s [130] construct of “somatic markers”. From the experimental point of view, a major contribution of the right AIC to the emotional experience was suggested by investigations conducted by Critchley et al. [131], Gray et al. [132] and Ogino et al. [133], which highlighted the importance of the right AIC in guiding second order “cognitive” representations of the bodily arousal state (see review in Gainotti [134]). A different interpretation of the contribution of the right and left AIC to the emotional experience was proposed by Craig [135], who assumed that hemispheric asymmetries at the insular level were based on an unequal representation of homeostatic activities, resulting from asymmetries in the peripheral autonomic nervous system. Due to these peripheral asymmetries, parasympathetic activities should be mainly represented in the left hemisphere, and sympathetic activities in the right hemisphere. A problem with this model is that in the forebrain of nonhuman primates there is little evidence of lateralized homeostatic afferent processing [136,137], whereas in humans there is strong evidence for the functional lateralization of homeostatic afferent pathways. However, if emotional functions are due to an asymmetrical central representation of homeostatic activities, and if the homeostatic afferents are lateralized only in humans, it should be predicted that a lateralization of emotional functions should only be observed in humans. This prediction is nevertheless disconfirmed by investigations that have documented the existence of continuities in emotion lateralization between humans and animals. This notion of a continuity in emotional lateralization between humans and (at least some kinds of) animals is also consistent with the observation that the putative neural substrate of the contribution of the right insular cortex to the emotional experience is also right lateralized both in humans and in nonhuman primates. Many authors, indeed, maintain that, from the neuroanatomical point of view, the greater role of the right AIC in emotional functions could be due to the prevalent right lateralization of a particular class of neurons (i.e., the “von Economo neurons” or “spindle neurons”), which project from the fronto-insular (FI) cortex to the anterior cingulate and the dorsolateral prefrontal cortex, relaying to these structures information related to autonomic control or awareness of homeostatic representations. The right lateralization of these neurons is important because Nimchinsky et al. [138] have discovered that “von Economo neurons” are present only in great apes and in humans (and also in big mammals, such as elephants [139]) and whales [140] but not in other primates, showing that these unique neurons appeared only recently during primate evolution. Furthermore, Allman et al. [141,142] have shown that the “Von Economo neurons” are as right lateralized in great apes as in humans, and Watkins et al. [143] have shown, in a large MRI study of adult humans, that AIC and the anterior cingulate gyrus (where these neurons are found) are significantly larger on the right than the left side. All these neuroanatomical findings are consistent with the hypothesis of a greater contribution of the right anterior insular cortex to the subjective experience of emotions both in humans and in great apes, but not in lower primates or in other animals.

## 5. A Provisional Interpretation of These Results

It is obviously very difficult to propose a general interpretation of these data. On one side, they suggest the existence of a continuum between humans and many animal species with respect to the issue of the right hemisphere prevalence in the modulation of sympathetic activities. On the other side, they suggest that a continuum may only exist between humans and great apes with respect to the asymmetric development of the emotional experience, thanks to the contribution of the right anterior insula to the processing and integration of the bodily afferent information. This conflict between these different accounts of the relations between human and animal asymmetries for emotional functions could, however, be mitigated by a weak version of the model assuming a continuum between humans and animal species on emotional lateralization. This weak version could consist in assuming that there is a common trend, but there could also be different steps in emotional lateralization. The continuum across many different animal species could be due to the fact that a lateralization of sympathetic functions (involved in brain and bodily activities necessary in emergency situations) is useful across these species, whereas different steps in the development of other asymmetries could be due to new needs, raised by the huge development of the brain and of its cognitive functions. This could explain why the right lateralization of sympathetic functions is consistent across many animal species, whereas asymmetries in emotional communication similar to those observed in humans have been documented only in primates and the right lateralization of the “von Economo neurons” involved in complex emotional functions, such as those concerning the subjective experience of emotions, has been reported only in humans and in great apes, but not in lower primates or in other animals.

In any case, the aim of this perspective article did not consist in opening a discussion on these provisional interpretations of some inconsistencies observed between human and animal studies on hemispheric asymmetries for emotions. Its goal rather consisted in stressing the fact that human and animal investigations should not proceed along parallel research streams with no or very few interactions, but should grow their efforts towards a necessary integration, increasing the openness to dialogue and the number of collaborative investigations. 
